# Supplementary material for: A new role for erythropoietin in the homeostasis of red blood cells
Source: Commun Biol. 2024 Jan 8;7:58. doi: 10.1038/s42003-023-05758-2 (PMC10774343; doi:10.1038/s42003-023-05758-2)
Supplement: Supplementary file 2 — Description of Additional Supplementary Files [file 42003_2023_5758_MOESM2_ESM.pdf]

Description of Additional Supplementary Files

File Name: Supplementary Data 1
Description: Experimental data shown in Figure 5.

File Name: Supplementary Data 2
Description: Jupyter notebooks with the code used for the numerical simulations shown in Figure 1.

File Name: Supplementary Software
Description: PDF version of the Jupyter notebooks with the code used for the numerical simulations of changes in red blood cell demand and hemmorhages shown in Figure 1.
