# Supplementary material for: A new role for erythropoietin in the homeostasis of red blood cells
Source: Commun Biol. 2024 Jan 8;7:58. doi: 10.1038/s42003-023-05758-2 (PMC10774343; doi:10.1038/s42003-023-05758-2)

Supplementary material for: ”A new role for erythropoietin in the homeostasis of red blood cells”

Clemente F. Arias[1,2,*], Nuno Valente-Leal[3], Federica Bertocchini[1], Sofia Marques[3], Francisco J. Acosta[4], and Cristina Fernandez-Arias[3,5,*]

[1]Centro de Investigaciones Biológicas (CSIC), Madrid, Spain
[2]Grupo Interdisciplinar de Sistemas Complejos (GISC), Madrid, Spain
[3]Instituto de Medicina Molecular, Universidade de Lisboa, Portugal
[4]Departamento de Ecología, Universidad Complutense de Madrid, Spain
[5]Departamento de Immunología, Facultad de Medicina, Universidad Complutense de Madrid, Spain

Corresponding authors:
[*]CFA: tifar@ucm.es
[*]CrF-A: crifer25@ucm.es

A. Code for the simulation of changes in cell demand

This notebook contains the code to run the numerical simulations of Model 2, given by the following equations:

$$\begin{cases} p'(t) = \lambda\big(K(t) - r(t)\big) \\ L'(t) = v(t) \\ r'(t) = p(t) - e^{-\mu L(t)} p\big(t - L(t)\big)\big(1 - L'(t)\big) - \mu r(t) \\ v'(t) = \rho\big(\bar{L} - L(t)\big) - \sigma L'(t) + \omega\big(K(t) - r(t)\big), \end{cases} \tag{1}$$

where r denotes the number of red blood cells (RBCs), $p(t)$ is the production of RBCs, L represents RBC lifespan, and v the rate of change of RBC lifespan. Parameters $\bar{L}$, μ, λ, ρ, σ, and ω are positive.

In particular, this file simulates the effect on the population of changes in cell demand

In [1]:

```
using DifferentialEquations
using Plots
```

Definition of the Delay Differential Equation (DDE) models

(Detailed information about implementing DDEs with fixed and variable delays in Julia can be found in https://docs.sciml.ai/DiffEqDocs/stable/tutorials/dde_example/)

In [2]:

```
h(p, t; idxs=nothing) = typeof(idxs) <: Number ? 1.0 : zeros(4) # Definition of history function h

function model_const(du,u,h,p,t)            # Model with constant lifespan (L'= 0 and v'= 0)
λ,μ,γ,L,ϱ,σ,ω = p

  hist = h(p, t-u[2]; idxs=1)

  du[1] = γ*(RBC_demand(t)-u[3])                                      # p'(t)
  du[2] = 0                                                           # L'(t)
  du[3] =  λ*u[1] -  λ*hist * exp(-μ*u[2]) - μ*u[3]     # r'(t)
  du[4] = 0                                                            # v'(t)
end

function model_var(du,u,h,p,t)              # Model with variable lifespan
  λ,μ,γ,L,ϱ,σ,ω = p

  hist = h(p, t-u[2]; idxs=1)

  du[1] = γ*(RBC_demand(t)-u[3])                                                   # p'(t)
  du[2] = u[4]                                                                     # Lifespan
  du[3] =  λ*u[1] - λ*hist * exp(-μ*u[2]) * (1 - du[2])  - μ*u[3]   # r'(t)
  du[4] = ϱ*(L - u[2])-σ * u[4]+ ω * du[1]                                       # v'(t)
end;
```

Definition of the DDE problems

Initial conditions and parameters

In [3]:

```
λ=1;
μ=0.0;
γ=0.0002;
L=100;
ϱ = 0.2;
```

```
σ = 20;
ω = 5;
p = (λ,μ,γ,L,ϱ,σ,ω);
tspan = (0.0,300001.0);
u0 = zeros(4)
u0[1]=0;
u0[2]=L;
u0[3]=0;
u0[4]=0;
```

DDEP problems

In [4]:

```
prob_var = DDEProblem(model_var,u0,h,tspan,p; dependent_lags = ((u,p,t) -> u[2],)); # Variable lifespan

prob_const = DDEProblem(model_const,u0,h,tspan,p; dependent_lags = ((u,p,t) -> u[2],)); # Constant lifespan

alg = MethodOfSteps(RK4());      # Numerical algorithm (MethodOfSteps solver)
```

Scenario 1

Definition of RBC demand

In [52]:

```
inc_demand =-.05;                # Change in cell demand expressed as a fraction of the population
veloc=.0125;                     # Velocity of the change in cell demand
```

In [53]:

```
function RBC_demand(t)           # To prevent issues with the initial population history,
                                 # simulations start with 0 cells.
  if t < 200000                  # The population grows to the desired size (in this case 100.000 cells).
    100000 - 100000/exp(0.0001*t)
  else
    100000+100000*inc_demand - (100000*inc_demand)/exp(veloc*(t-200000))  # Changes in cell demand are simulated
    end                                             # at time t = 200.000, once the population has stabilizeds
end
```

Out[53]: RBC_demand (generic function with 1 method)

In [54]:

```
plot(RBC_demand,199900,201000,size=(500,200),legend=false)
```

Out[54]:

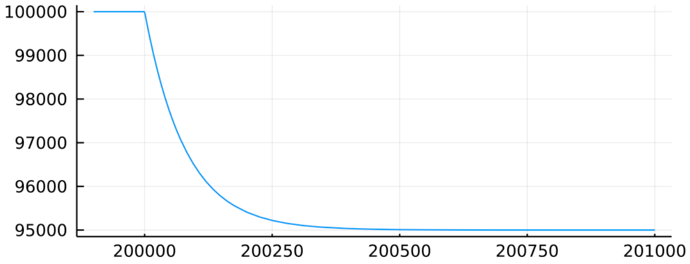

Numerical simulations

In [55]:

```
sol_const = solve(prob_const,alg,maxiters = 1e7); # Constant lifespan
```

In [56]:

```
sol_var = solve(prob_var,maxiters = 1e7,alg); # Variable lifespan
```

Results

In [57]:

```
# Number of cells (Variable lifespan)
plot(sol_var.t.-199990,sol_var[3,:],
    size = (300, 200),legend=false,xlims=(0,1100),ylims=(90000,105000))
```

Out[57]:

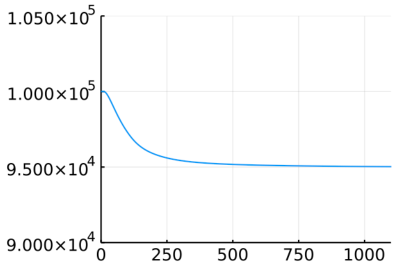

In [58]:

```
# Number of cells (Constant lifespan)
plot!(sol_const.t.-199990,sol_const[3,:],
    size = (300, 200),legend=false,xlims=(0,1100),ylims=(90000,108000))
```

Out[58]:

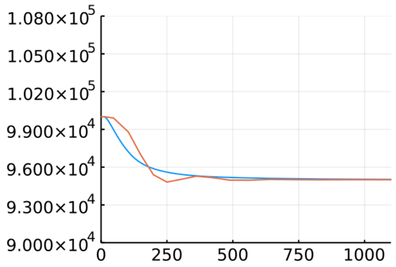

In [59]:

```
# RBC production (Variable lifespan)
plot(sol_var.t.-199990,sol_var[1,:],
    size = (300, 200),legend=false,xlims=(0,500))
```

Out[59]:

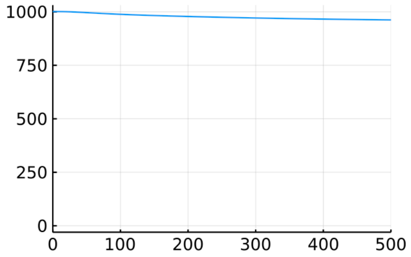

In [60]:

```
# RBC production (Constant lifespan)
plot(sol_const.t.-199990,sol_const[1,:],
    size = (300, 200),legend=false,xlims=(0,500))
```

Out[60]:

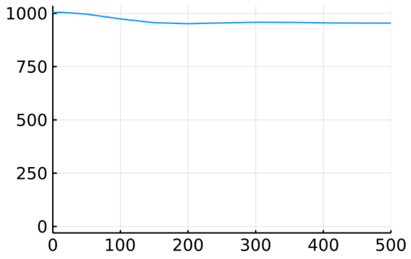

In [61]:

```
# Variable lifespan
plot(sol_var.t.-199990,sol_var[2,:],
    size = (300, 200),legend=false,xlims=(10,28))
```

Out[61]:

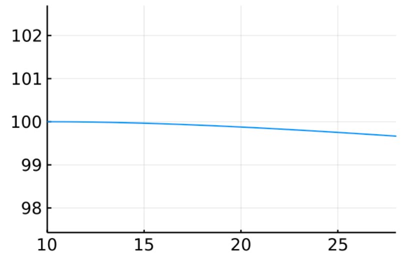

Scenario 2

Definition of RBC demand

In [15]:

```
function RBC_demand(t)
    if t < 200000
        100000 - 100000/exp(0.0001*t)
    else
        100000+100000*.1*sin(.01*(t - 200000))
    end
end
```

Out[15]: RBC_demand (generic function with 1 method)

In [16]:

```
plot(RBC_demand,199900,201000,size=(300,200),legend=false)
```

Out[16]:

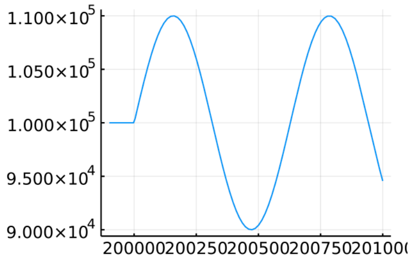

Numerical simulations

In [17]:

```
sol_const = solve(prob_const,alg,maxiters = 1e7); # Constant lifespan
```

In [18]:

```
sol_var = solve(prob_var,maxiters = 1e7,alg); # Variable lifespan
```

Results

In [47]:

```
# Number of cells (Variable lifespan)
plot(sol_var.t.-199990,sol_var[3,:],
    size = (300, 200),legend=false,xlims=(0,1000),ylims=(85000,115000))
```

Out[47]:

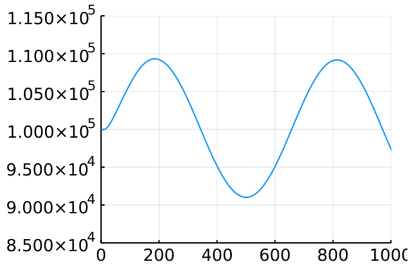

In [48]:

```
# Number of cells (Constant lifespan)
plot!(sol_const.t.-199990,sol_const[3,:],
    size = (300, 200),legend=false,xlims=(0,1000),ylims=(85000,115000))
```

Out[48]:

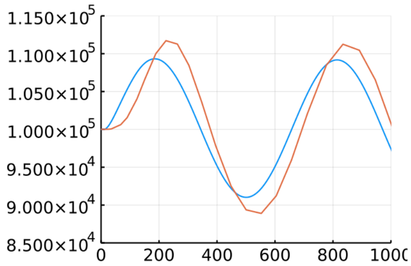

In [49]:

```
# RBC production (Variable lifespan)
plot(sol_var.t.-199990,sol_var[1,:],
    size = (300, 200),legend=false,xlims=(0,1000),ylims=(0,1200))
```

Out[49]:

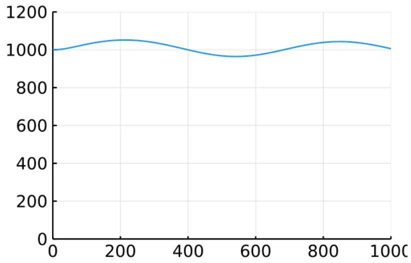

In [50]:

```
# RBC production (Constant lifespan)
plot(sol_const.t.-199990,sol_const[1,:],
    size = (300, 200),legend=false,xlims=(0,1000),ylims=(0,1200))
```

Out[50]:

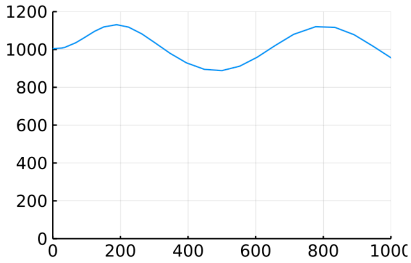

In [51]:

```
# Variable lifespan
plot(sol_var.t.-199990,sol_var[2,:],
    size = (300, 200),legend=false,xlims=(0,1000),ylims=(90,110))
```

Out[51]:

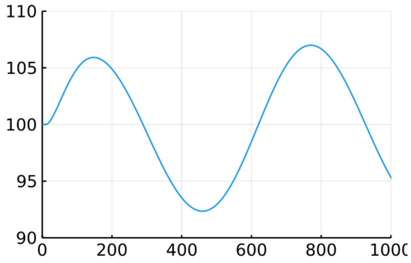

B. Code for the simulation of hemorrhages

This notebook contains the code to run the numerical simulations of Model 2, given by the following equations:

$$\begin{cases} p'(t) = \lambda\big(K(t) - r(t)\big) \\ L'(t) = v(t) \\ r'(t) = p(t) - e^{-\mu L(t)} p\big(t - L(t)\big)\big(1 - L'(t)\big) - \mu r(t) \\ v'(t) = \rho\big(\bar{L} - L(t)\big) - \sigma L'(t) + \omega\big(K(t) - r(t)\big), \end{cases}$$

where r denotes the number of red blood cells (RBCs), $p(t)$ is the production of RBCs, L represents RBC lifespan, and v the rate of change of RBC lifespan. Parameters $\bar{L}$, μ, λ, ρ, σ, and ω are positive.

In particular, this file simulates the effect of hemorrhages on the population dynamics

In [1]:

```
using DifferentialEquations
using Plots
```

Definition of the Delay Differential Equation (DDE) models

(Detailed information about implementing DDEs with fixed and variable delays in Julia can be found in https://docs.sciml.ai/DiffEqDocs/stable/tutorials/dde_example/)

In [2]:

```
h(p, t; idxs=nothing) = typeof(idxs) <: Number ? 1.0 : zeros(4) # Definition of history function h

hem_death = 0.9                           # Fraction of RBCs lost in hemorrhage (value between 0 and 1)

function coef_Hem(t,L)                    # This function is used to prevent RBCs lost in hemorrhage to
    if t>200000 && t< 200000+L            # die again from old age when they reach their lifespan
      hem_death
    else
        1.
    end
end

function model_const(du,u,h,p,t)           # Model with constant lifespan (L'= 0 and v'= 0)
λ,μ,γ,L,ϱ,σ,ω = p

  hist = h(p, t-u[2]; idxs=1)

  du[1] = γ*(RBC_demand(t)-u[3])                                        # p'(t)
  du[2] = 0                                                             # L'(t)
  du[3] =  λ*u[1] - coef_Hem(t,u0[2])* λ*hist * exp(-μ*u[2]) - μ*u[3]     # r'(t)
  du[4] = 0                                                              # v'(t)
end

function model_var(du,u,h,p,t)             # Model with variable lifespan
  λ,μ,γ,L,ϱ,σ,ω = p

  hist = h(p, t-u[2]; idxs=1)

  du[1] = γ*(RBC_demand(t)-u[3])                                                     # p'(t)
  du[2] = u[4]                                                                       # Lifespan
  du[3] =  λ*u[1] -coef_Hem(t,u0[2])* λ*hist * exp(-μ*u[2]) * (1 - du[2])  - μ*u[3]    # r'(t)
  du[4] = ϱ*(L - u[2])-σ * u[4]+ ω * du[1]                                         # v'(t)
end;
```

Definition of hemorrhages

In [3]:
```
function condition_hem(u,t,integrator)            # Hemorrhage occurs at time t = 200000
  t-200000.0<0
end;

affect_hem! = (integrator -> integrator.u[3] = hem_death*integrator.u[3]); # It entails the sudden loss
                                                                           # of a fraction of RBCs, given by
                                                                           # parameter hem_death defined above

ccb_hem = ContinuousCallback(condition_hem,affect_hem!);

cbs_hem= CallbackSet(ccb_hem);
```

Definition of the DDE problems

Initial conditions and parameters

In [4]:
```
λ=1;
μ=0.0;
γ=0.0002;
L=100;
ϱ = 0.2;
σ = 20;
ω = 5;
p = (λ,μ,γ,L,ϱ,σ,ω);
tspan = (0.0,300001.0);
u0 = zeros(4)
u0[1]=0;
u0[2]=L;
u0[3]=0;
u0[4]=0;
```

DDEP problems

In [5]:
```
prob_var = DDEProblem(model_var,u0,h,tspan,p; dependent_lags = ((u,p,t) -> u[2],)); # Variable lifespan

prob_const = DDEProblem(model_const,u0,h,tspan,p; dependent_lags = ((u,p,t) -> u[2],)); # Constant lifespan

alg = MethodOfSteps(RK4());      # Numerical algorithm (MethodOfSteps solver)
```

Inizialization of the population

In [6]:
```
function RBC_demand(t)              # To prevent issues with the population history, simulations start with 0 cells.
    100000 - 100000/exp(0.0001*t)   # The population grows progressively up to the desired value
end;                                # (in this case 100.000 cells)
```

In [7]:
```
plot(RBC_demand,0,250000,size=(500,200),legend=false)    # hemorrhages are simulated at time t=200.000, once the
                                                         # population has stabilised
```

Out[7]:

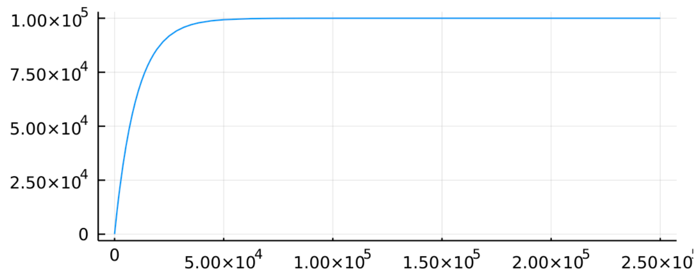

Numerical simulations

In [8]:
```
@time sol_const = solve(prob_const,alg,maxiters = 1e7,callback=cbs_hem); # Constant lifespan
```

In [9]:
```
@time sol_var = solve(prob_var,maxiters = 1e7,alg,callback=cbs_hem); # Variable lifespan
```

Results

In [10]:
```
# Number of cells (Variable lifespan)
plot(sol_var.t.-sol_var.t[findall(x -> x > 200000, sol_var.t)][1],sol_var[3,:],
    size = (300, 200),legend=false,xlims=(0,1100),ylims=(90000,105000))
```

Out[10]:

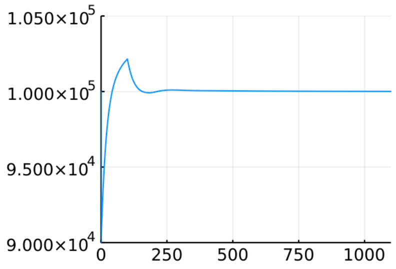

In [11]:
```
# Number of cells (Constant lifespan)
plot!(sol_const.t.-sol_const.t[findall(x -> x > 200000, sol_const.t)][1],sol_const[3,:],
    size = (300, 200),legend=false,xlims=(0,1100),ylims=(90000,108000))
```

Out[11]:

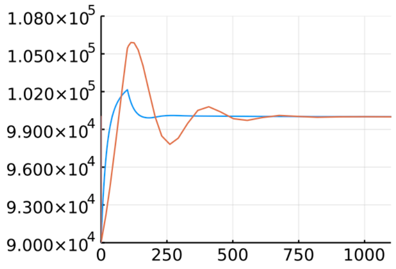

In [12]:
```
# RBC production (Variable lifespan)
plot(sol_var.t.-sol_var.t[findall(x -> x > 199900, sol_var.t)][1],sol_var[1,:],
    size = (300, 200),legend=false,xlims=(0,500))
```

Out[12]:

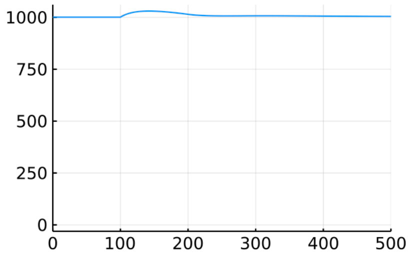

In [13]:
```
# RBC production (Constant lifespan)
plot(sol_const.t.-sol_const.t[findall(x -> x > 199900, sol_const.t)][1],sol_const[1,:],
    size = (300, 200),legend=false,xlims=(0,500))
```

Out[13]:

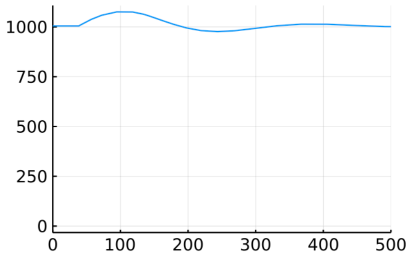

In [14]:

```
# Variable lifespan
plot(sol_var.t.-sol_var.t[findall(x -> x > 199990, sol_var.t)][1],sol_var[2,:],
    size = (300, 200),legend=false,xlims=(10,28))
```

Out[14]:

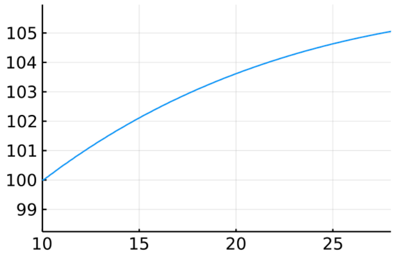

Supplement: Supplementary file 5 — Supplementary Software [file 42003_2023_5758_MOESM5_ESM.pdf]
